# Can active sun exposure decrease the risk of giant cell arteritis and polymyalgia rheumatica in women?

**DOI:** 10.1093/rap/rkad071

**Published:** 2023-08-18

**Authors:** Karl Gisslander, Raïssa de Boer, Christian Ingvar, Carl Turesson, Karolin Isaksson, David Jayne, Aladdin J Mohammad

**Affiliations:** Rheumatology, Department of Clinical Sciences Lund, Lund University, Lund, Sweden; Rheumatology, Department of Clinical Sciences Lund, Lund University, Lund, Sweden; Surgery, Department of Clinical Sciences, Lund University, Lund, Sweden; Rheumatology, Department of Clinical Sciences Malmö, Lund University, Malmö, Sweden; Surgery, Department of Clinical Sciences, Lund University, Lund, Sweden; Department of Surgery, Kristianstad Hospital, Kristianstad, Sweden; Department of Medicine, University of Cambridge, Cambridge, UK; Rheumatology, Department of Clinical Sciences Lund, Lund University, Lund, Sweden; Department of Medicine, University of Cambridge, Cambridge, UK

**Keywords:** GCA, PMR, sun exposure, environmental risk factor

## Abstract

**Objectives:**

To study if active sun exposure among women affects the risk of developing GCA or PMR in a prospective cohort study with restricted latitudinal variability.

**Methods:**

We linked the response to questions relating to sun exposure from the Melanoma Inquiry in Southern Sweden (MISS) prospective cohort study in women to the risk of developing GCA or PMR. Healthcare data were gathered from the Skåne Healthcare Register (SHR), covering all public healthcare consultations. The direct effect of active sun exposure on the risk of developing GCA or PMR was assessed using Cox proportional hazards models adjusted for covariates based on a directed acyclic graph.

**Results:**

A total of 14 574 women were included in the study; 601 women were diagnosed with GCA or PMR (144 and 457, respectively) during the follow-up time. Women with moderate or high sun exposure were not less likely to develop GCA or PMR compared with women that indicated they avoided sun exposure [hazard ratio (HR) 1.2 (CI 0.9, 1.6) and 1.3 (0.9, 1.9), respectively] when adjusted for diabetes, hyperlipidaemia, hypertension, smoking, obesity and stratified by age. Similar patterns were observed when studying only GCA [HR 1.2 (CI 0.7, 2.3) and 1.3 (0.7, 2.6)] and only PMR [HR 1.3 (CI 0.9, 1.8) and 1.4 (0.9, 2.0)].

**Conclusion:**

Active sun exposure did not affect the risk of developing GCA or PMR in women in a cohort with restricted latitudinal variability.

Key messages

- Active sun exposure does not decrease the risk of developing GCA or PMR.
- Geographic variation in the incidence of GCA and PMR may be explained by other factors.

## Introduction

GCA is an inflammatory disease affecting large and medium-sized arteries. GCA is frequently associated with PMR, an inflammatory condition causing proximal bilateral pain and stiffness of the extremities. GCA and PMR occur almost exclusively in individuals ≥50 years of age and the incidence rates for this age group in Scandinavian populations are approximately 20 and 50 per 100 000, respectively [1, 2]. These conditions may occur simultaneously or in isolation. Approximately 50% of patients with GCA present with PMR before, at the time of or after the diagnosis of GCA [3]. Both GCA and PMR are diseases that may have complex and non-specific symptoms, which hampers a timely and accurate diagnosis. Yet, early treatment of GCA is essential in preventing severe outcomes such as blindness, stroke, aortic aneurysm, dissection and rupture [4–7]. A better understanding of the risk factors for GCA and PMR may decrease diagnostic delay or even allow for preventative measures. Recent data suggest that circulating cytokines related to T cell activation may be elevated years before clinical onset of GCA [8]. However, the aetiology of these diseases remains poorly understood and seems to be a combination of a genetic predisposition and environmental factors [9, 10]. Latitudinal and seasonal variation in the incidence of GCA has been reported [9, 11] similar to other autoimmune disorders [12]. There is also geographic variation in PMR incidence, with higher estimates from northern Europe and North America [13]. A latitudinal and seasonal variation suggests sun exposure as an environmental factor that may be of major importance in developing these conditions. However, this is poorly understood due to genetic factors confounding with latitudinal variation [10] and conflicting results between studies [11].

Sun exposure is closely related to human health, with perhaps the most well-known effect being sunburn and its association with skin cancer [14]. However, it has become increasingly clear that sun exposure is beneficial and that a lack of sun exposure is related to a plethora of health concerns, including cardiovascular disease, cancer and various autoimmune diseases [15]. Sun exposure is the major source of vitamin D in many populations and it is often assumed that this is the primary mechanism for the beneficial effects of sunlight. Sun exposure can benefit health via pathways independent of vitamin D [16–18]. Vitamin D levels are not necessarily causative of the association between latitudinal variation in ultraviolet (UV) exposure and autoimmune or other diseases [19–21]. Moreover, sun exposure has changed tremendously in modern society compared with our ancestors. Nowadays, work takes place predominantly indoors, minimizing occupational sun exposure. In addition, the emergence of long-distance travel has made going on holidays to sunny locations widely available. These factors, among other things, have led to sun exposure being increasingly the result of interindividual differences and not merely latitudinal variation in UV exposure [20, 22]. This is also highlighted by the lack of a clear latitudinal gradient in vitamin D levels [23], whereas vitamin D levels among individuals living in the same latitude can vary substantially due to behavioural differences [24–26]. To gain more insight into the effect of sun exposure on the risk of GCA and PMR, we focus on interindividual variations in sun exposure.

In this study we address whether different levels of sun exposure due to variations in behaviour could affect the risk of developing GCA and PMR. By focusing on a cohort in southern Sweden (≈56° N latitude), we investigate the effect of sun exposure while bypassing the issue of confounding factors with latitudinal gradients. We hypothesize that variations in sun exposure due to interindividual differences in behaviour affect the risk of developing these diseases. Specifically, we predict that women with greater sun exposure due to sunbathing in summer, going on winter or summer sun holidays and/or the use of indoor tanning have a lower risk of developing GCA or PMR compared with women who avoid sun exposure.

## Methods

In this study we link the responses to questions relating to sun exposure in a prospective survey with the risk of subsequently developing GCA or PMR in women living in Skåne (Scania) the southernmost province of Sweden. Skåne covers an area of 11 035 km^2^ (≈3% of the total area of Sweden). In 2019 it had 1 389 336 inhabitants (13% of the total Swedish population). The population ≥50 years of age was 512 867, with the foreign-born population in that age group being 19.5%.

### Questionnaire

The Melanoma Inquiry in Southern Sweden (MISS) was a prospective cohort study looking at risk factors for melanoma [27]. The study started in 1990 by sending out a questionnaire to close to 40 000 women in southern Sweden. The questionnaire included various questions regarding sun exposure, such as the habit of sunbathing in summer and going on holidays. Here we here focus on the 24 106 women who responded to the follow-up questionnaire that was conducted between 2000 and 2002. This was done so that the time of the questionnaire coincided with the available medical data. All participating women gave written informed consent.

### The Skåne Healthcare Register (SHR)

Healthcare data was gathered from the SHR, which covers all public healthcare consultations from primary care through hospitalization in Skåne [28]. We gathered the date on death and codes of the International Classification of Disease, 10th revision (ICD-10), that were assigned by physicians between 1998 and 2019 from the SHR.

### Sun exposure

Our estimation of sun exposure was based on responses to the MISS questionnaire. Sun exposure was categorized following previous studies [29, 30] and was based on four questions that concerned the frequency of sunbathing in summer (never, 1–14 times, 15–30 times, >30 times), going on holiday to sunbathe in the winter (no, 0–3 sun days, 4–10 sun days, >10 sun days) or in summer (never, every other year, every year, more than once a year) and the use of indoor tanning (never, 0–3 times/year, 4–10 times/year, >10 times/year). The participants’ responses to these questions were dichotomized into ‘ever’ or ‘never’. The number of questions replied to with ‘ever’ determined whether a participant was regarded as low sun exposure (none of the questions replied to with ‘ever’), moderate sun exposure (1–2 questions replied to with ‘ever’) and high sun exposure (3–4 questions replied to with ‘ever’).

### GCA and PMR

We identified women with one or more ICD-10 code for GCA [GCA with PMR (M315) or other GCA (M316)] or PMR (M353) in the SHR (Supplementary Table S1, available at *Rheumatology Advances in Practice* online). Patients were classified according to the first registration of one of these diagnoses. Only ICD-10 codes assigned after the date of the MISS questionnaire and after the age of 50 were considered. The start date of the study was considered the age of 50 or the date of answering the MISS questionnaire, whichever happened last. The ‘time at risk’ was calculated from the start date to the date of GCA or PMR diagnosis, date of death or end of follow-up, whichever came first. The end of follow-up was defined as the last recorded healthcare consultation in the SHR. This was done to account for people who moved away from Skåne before the end of the study or were lost from the registry for other reasons. Medical history was available until 31 December 2019. This marks the end of the study.

### Covariates

A range of cardiovascular risk factors has been suggested in the development of GCA. Smoking and hypertension may increase the risk of developing GCA [31, 32], while diabetes, hyperlipidaemia and obesity may have a protective effect [33–36]. It is well established that the risk of GCA increases with age. Sun exposure may have an influence on the risk of hypertension and diabetes [17, 37]. We identified women with an ICD-10 code for hypertension (I10), hyperlipidaemia (E78) or diabetes (E10-E11) assigned before the start date of the study or during the time at risk (Supplementary Table S1, available at *Rheumatology Advances in Practice* online). In addition, we obtained information from the MISS questionnaire on age at the time of filling out the questionnaire, smoking (ever or never) and obesity (obese: BMI >30, not obese: BMI ≤30 kg/m^2^).

### Statistical analyses

All statistical analyses were performed in RStudio version 2022.12.0 (Posit Software, Boston, MA, USA). The risk of developing GCA or PMR in relation to sun exposure was analysed using Cox proportional hazards models using the ‘coxph’ function in the ‘survival’ package [38]. The time variable was the time at risk (see above). The diagnosis of GCA or PMR was included as the binary response variable (0: person was not assigned an ICD-10 code for GCA or PMR, 1: person was assigned one or more ICD-10 codes for GCA or PMR). Sun exposure was the main predictor variable.

**Figure 1.**
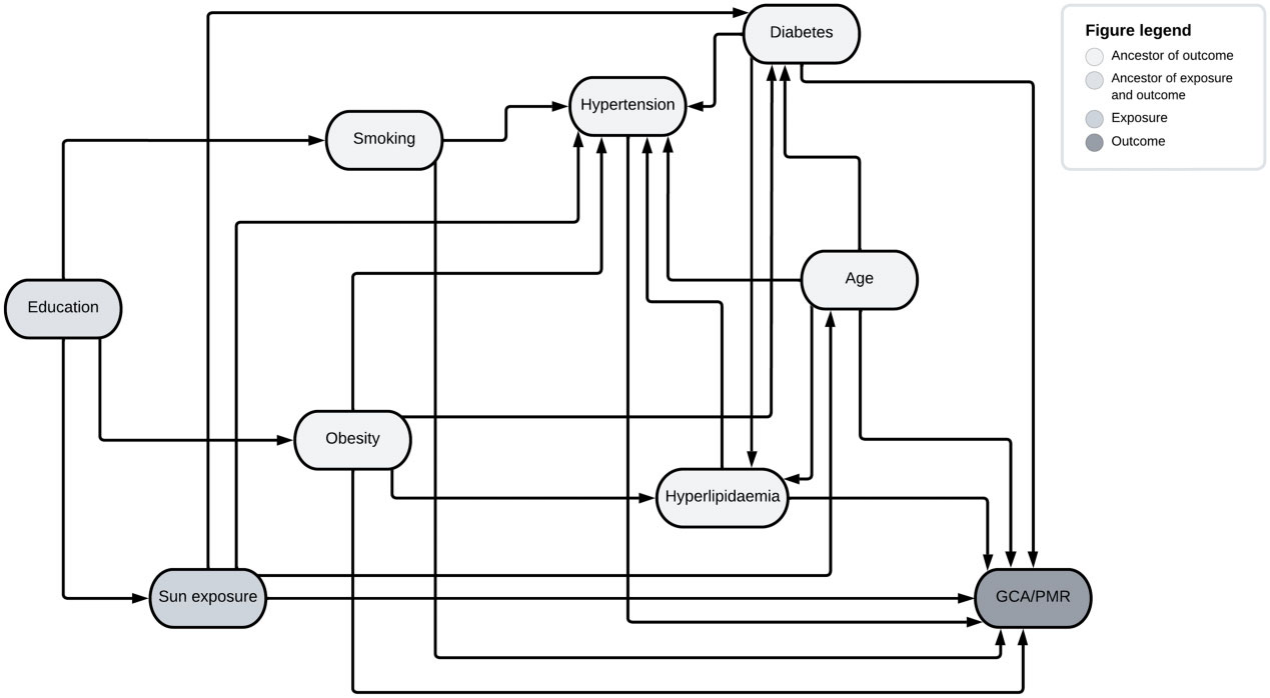
Directed acyclic graph showing the causal relationship between covariates, exposure and outcome. Minimal sufficient adjusted sets for estimating the direct effect of sun exposure on GCA/PMR: age, diabetes, hyperlipidaemia, hypertension, obesity and smoking

To estimate the direct effect of sun exposure on the outcome GCA or PMR, multivariable models were constructed with sun exposure and covariates based on assumptions from a directed acyclic graph using the ‘dagitty’ package [39] (Fig. 1). A multivariable Cox proportional hazards model including hypertension, hyperlipidaemia, diabetes, age, smoking and obesity was built. Cases with missing data for any of the model variables were excluded from the analysis. Subanalyses using the same covariates were performed, estimating the direct effect of sun exposure on the sole outcomes GCA or PMR. A sensitivity analysis was performed, requiring a minimum of two ICD-10 codes for GCA or PMR acquired at separate visits. A diagnosis of hypertension, hyperlipidaemia or and diabetes after the start date was considered as time-dependent covariates.

Model assumptions were checked using the multivariable model. First, we evaluated the assumption of proportional hazards being constant over time for each covariate via graphical inspection of Schoenfeld residuals. This demonstrated that the effect of age decreased with longer follow-up time. We therefore added age as a stratified variable in the model. Second, we checked for potential influential observations by specifying residuals to be of type ‘dfbeta’, which shows the expected change in the regression coefficient after deleting each observation one by one. No problems regarding influential data points were identified. We did not test for non-linearity given that all factors were categorical. Results are presented as hazard ratios (HRs) with 95% CIs and visualized as forest plots or tables. The Ethics Committee of Lund University approved the study (Dnr. LU 34-92 and 849-2005).

## Results

**Figure 2.**
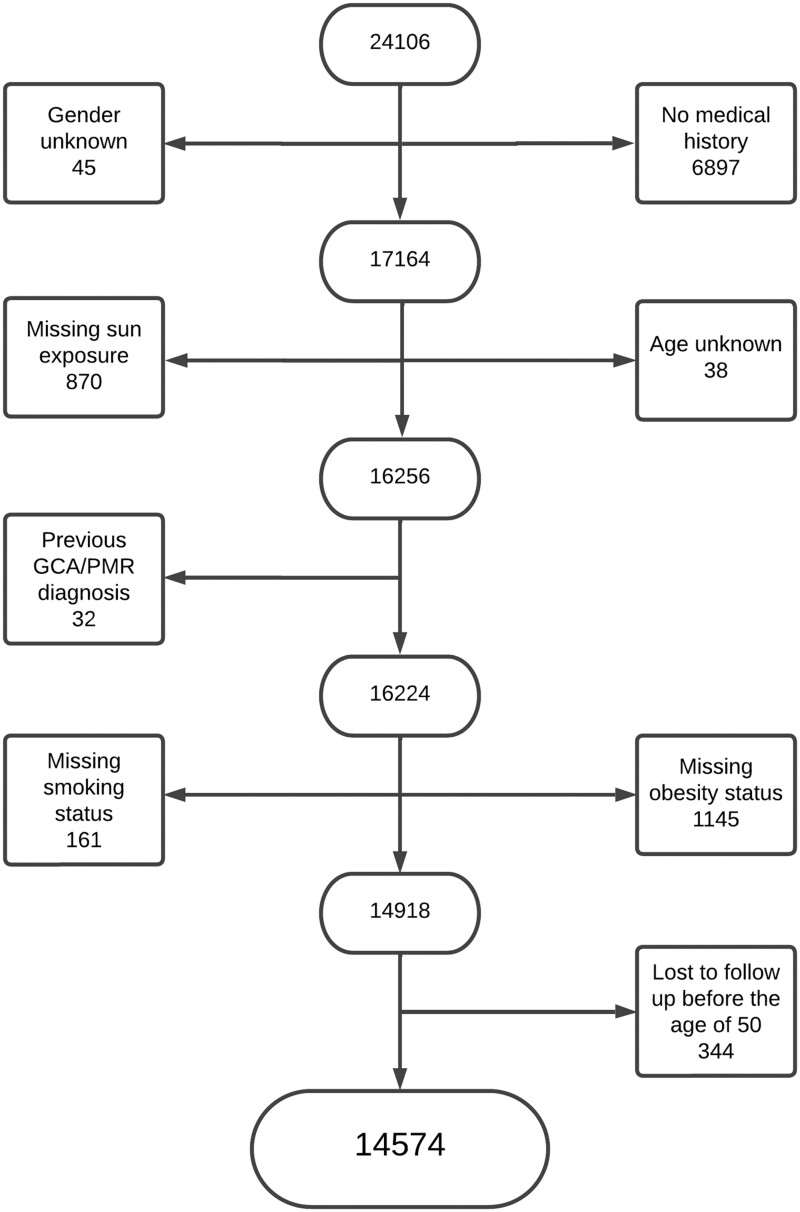
Exclusion flow chart for ‘Can active sun exposure decrease the risk of GCA and PMR in women?’

A total of 14 574 women were included in this study. Reasons for exclusion from the study are shown in Fig. 2. Descriptive data for the cohort stratified by sun exposure level can be seen in Table 1. The majority of women fell in the moderate [*n* = 8851 (61%)] and greatest sun exposure categories [*n* = 4728 (32%)], while few avoided sun exposure [*n* = 995 (7%)]. Of these, 601 women (4%) were subsequently diagnosed with GCA or PMR (144 and 457, respectively). The median time to diagnosis from inclusion in the study was 11.2 years (interquartile range 6.7–14.6). In this subset, 46 (8%) avoided sun exposure, 388 (65%) had moderate sun exposure and 167 (28%) fell into the greatest sun exposure category. The distribution of baseline features differed across the three exposure levels, with the low sun exposure group being older, having a higher BMI, fewer smokers, more diabetes and more hypertension. The frequency of hyperlipidaemia was similar across the sun exposure levels (Table 1).

**Table 1.** Baseline features, outcomes and follow-up times stratified by sun exposure level

Characteristics	Sun exposure		
	Low	Moderate	High
Age (years), *n* (%)			
50–54	242 (24)	4133 (47)	2976 (63)
55–59	152 (15)	1384 (16)	832 (18)
60–64	172 (17)	1243 (14)	479 (10)
65–69	205 (21)	1185 (13)	280 (6)
70–75	224 (23)	906 (10)	161 (3)
Smoker, *n* (%)	491 (49)	4894 (55)	2942 (62)
BMI >30 kg/m^2^, *n* (%)	216 (22)	1093 (12)	316 (7)
Diabetes^a^, *n* (%)	162 (16)	1025 (12)	369 (8)
Hypertension^a^, *n* (%)	559 (56)	4191 (47)	1870 (40)
Hyperlipidaemia^a^, *n* (%)	215 (22)	1985 (22)	976 (21)
Outcome, *n* (%)			
PMR	35 (4)	295 (3)	127 (3)
GCA	11 (1)	93 (1)	40 (1)
Follow-up time, years, mean (s.d.)	14.2 (5.5)	14.2 (5.4)	13.7 (5.4)

a Accumulated during follow-up time.

**Figure 3.**
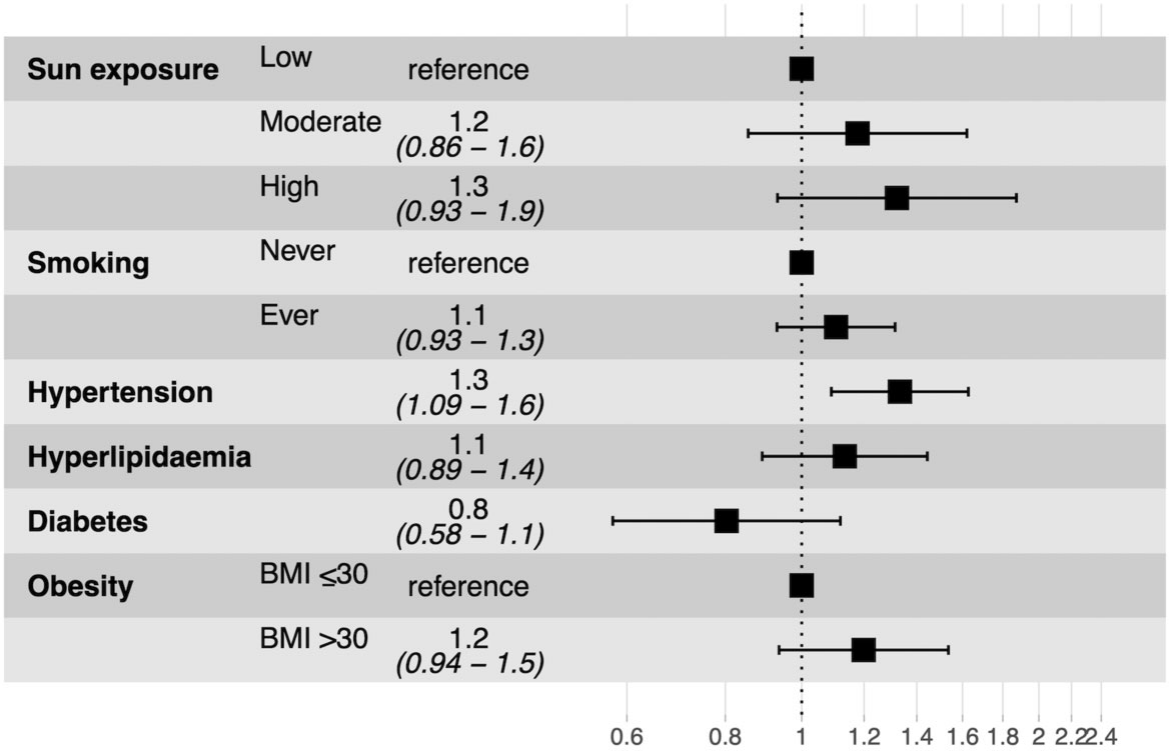
HRs and 95% CIs for the development of GCA/PMR of variables included in the multivariable model. Age was included as a stratified variable but not shown. Hypertension, hyperlipidaemia and diabetes were included as time-dependent variables

Women with moderate or high sun exposure were not less likely to develop GCA or PMR compared with women who indicated they avoided sun exposure [HR 1.2 (CI 0.9, 1.6) and 1.3 (0.9, 1.9), respectively] when adjusted for diabetes, hyperlipidaemia, hypertension, smoking, obesity and stratified by age (Fig. 3).

A univariable model showed that the risk of developing GCA or PMR was clearly associated with increasing age in a dose–response relationship (Table 2). We saw an association with developing GCA/PMR and hyperlipidaemia, hypertension and obesity, but not with sun exposure, diabetes or smoking (Table 2).

**Table 2.** Univariable HRs and 95% CIs of the risk of developing of GCA/PMR, GCA or PMR

Variables	GCA/PMR, HR (95% CI)	GCA, HR (95% CI)	PMR, HR (95% CI)
Age (years)			
50–54	REF	REF	REF
55–59	2.0 (1.5, 2.6)	2.1 (1.3, 3.5)	2.1 (1.7, 2.9)
60–64	2.4 (1.8, 3.2)	2.0 (1.2, 3.5)	2.7 (2.0, 3.6)
65–69	3.7 (2.8, 4.8)	2.7 (1.6, 4.6)	4.1 (3.1, 5.5)
70–75	4.5 (3.4, 5.9)	4.3 (2.6, 7.1)	4.8 (3.5, 6.5)
Smoker			
Never	REF	REF	REF
Ever	0.9 (0.8, 1.1)	0.6 (0.4, 0.8)	1.0 (0.8, 1.2)
Sun exposure			
Low	REF	REF	REF
Moderate	0.9 (0.7, 1.3)	1.0 (0.5, 1.8)	1.0 (0.7, 1.4)
High	0.8 (0.6, 1.1)	0.8 (0.4, 1.6)	0.8 (0.6, 1.2)
Obesity			
BMI ≤30 kg/m^2^	REF	REF	REF
BMI >30 kg/m^2^	1.3 (1.0, 1.7)	1.2 (0.7, 1.9)	1.4 (1.1, 1.8)
Time dependent			
Hypertension	1.7 (1.4, 2.1)	1.2 (0.9, 1.8)	1.7 (1.4, 2.1)
Hyperlipidaemia	1.4 (1.1, 1.8)	1.2 (0.7, 1.9)	1.4 (1.1, 1.7)
Diabetes	1.1 (0.8, 1.5)	1.0 (0.5, 1.8)	1.2 (0.9, 1.7)

REF: reference level.

In subanalyses we did not see an effect of sun exposure on the risk of developing the sole outcome of GCA [HR 1.2 (CI 0.7, 2.3) and 1.3 (0.7, 2.6)] nor the sole outcome of PMR [HR 1.3 (CI 0.9, 1.8) and 1.4 (CI 0.9, 2.0)] for moderate and high sun exposure, respectively, when adjusted for diabetes, hyperlipidaemia, hypertension, smoking, obesity and stratified by age (Table 3).

**Table 3.** Multivariable HRs and 95% CIs of the risk of developing GCA or PMR, adjusted for diabetes, hyperlipidaemia, hypertension, smoking, obesity and stratified by age^a^

Variables	GCA, HR (95% CI)	PMR, HR (95% CI)
Sun exposure		
Low	REF	REF
Moderate	1.2 (0.7, 2.3)	1.3 (0.9, 1.8)
High	1.3 (0.7, 2.6)	1.4 (0.9, 2.0)
Obesity		
BMI ≤30 kg/m^2^	REF	REF
BMI, >30 kg/m^2^	1.1 (0.7, 1.8)	1.2 (1.0, 1.6)
Smoker		
Never	REF	REF
Ever	0.7 (0.5, 0.9)	1.2 (1.0, 1.4)
Time dependent		
Hypertension	1.1 (0.7, 1.6)	1.4 (1.1, 1.7)
Hyperlipidaemia	1.1 (0.7, 1.8)	1.1 (0.9, 1.4)
Diabetes	0.8 (0.4, 1.6)	0.9 (0.7, 1.3)

REF: reference level.

a Age was included as a stratified variable but not shown.

A sensitivity analysis requiring a minimum of two ICD-10 codes for GCA/PMR at different visits was performed, reducing the total number of subjects with a GCA/PMR diagnosis to 492 (93 and 399, respectively), with no effect on the main results (Supplementary Tables S2–S4, available at *Rheumatology Advances in Practice* online).

## Discussion

In this study we found no evidence that variation in sun exposure among women living in Skåne affected the risk of developing GCA or PMR. In contrast to our prediction, women who indicated they regularly sought sun exposure, by going on sun holidays, sunbathing in summer or using indoor tanning, were not less likely to develop GCA or PMR compared with women who indicated they never actively sought sun exposure.

It could be that the variation in sun exposure was too small to reveal any significant differences in the risk of developing GCA/PMR or that there were too few women diagnosed with GCA or PMR to obtain sufficient power to detect a perhaps small effect, a common issue with studying rare diseases. The slope of the HRs indicates a dose–response relationship with a decrease in the risk of GCA/PMR with lower sun exposure, although it was not statistically significant.

Previous studies on the MISS cohort showed that women with high sun exposure had a 2-fold reduced mortality rate [29] and a lower risk of venous thromboembolisms [40] and type 2 diabetes mellitus [37] compared with women who avoided sun exposure. This indicates that the variation in sun exposure should be large enough to detect differences in the development of GCA/PMR instead indicating a potential power issue. The percentages developing GCA or PMR during the follow-up time were low (1% and 3%, respectively).

It is known that the potential risk factors for developing GCA and PMR may influence each other in a complex pattern. To adjust for confounding effects and to estimate the direct effect of sun exposure on the outcome GCA or PMR we built the multivariable model based on assumptions from a directed acyclic graph. This model also adjusted for any differences seen in baseline features among the sun exposure levels. However, there are suspected risk factors not accounted for in the model. Most notably there is evidence of a genetic predisposition to GCA [10]. In our study the genetic background of the cohort was not investigated and could not be adjusted for. However, as the study included women ≥50 years of age from a defined geographic area, genetic variability is likely to be limited.

Sun exposure is the major source of vitamin D and it is therefore often assumed that this is the primary mechanism for the beneficial effects of sunlight. Vitamin D has immunomodulatory effects, including downregulation of Th1 and Th17 cells and upregulation of Treg cells [41–43]. Th1 and Th17 cell linages are crucial for initiating and maintaining arterial inflammation, while the levels of Treg cells are reported to be low in GCA [44]. However, the timing of a potential sun environmental trigger in the aetiology of GCA or PMR is not known. It has previously been suggested that the incidence of GCA in southern Sweden is highest in spring [45], potentially indicating a symptom debut after a period of low sun exposure. In our study, behaviour towards sun exposure was measured at a single point in time and not through repeated measuring. We do not know if the intra-individual attitude towards sun exposure changed over time and we do not have data on the use of supplemental vitamin D. Furthermore, the correlation of sun exposure level assigned in the questionnaire and the actual vitamin D status is unknown. The major emission spectrum of indoor tanning, included in the sun exposure definition, does not effectively induce vitamin D production [46].

The hypothesized effect of sun exposure on the development of inflammatory rheumatic disease or vasculitis is not unique for PMR and GCA. In ANCA-associated vasculitis (AAV), the effect of sun exposure has been a long-standing debate. Observations of latitudinal differences in disease phenotype [47] have led to several studies investigating the effect of latitude and estimated UV exposure in disease onset, relapse, phenotypic and serologic expression [12, 48, 49]. The available evidence suggests an increased risk of granulomatosis with polyangiitis and proteinase 3 antibody positivity with increasing latitude and decreased UV exposure, with an inverse relationship for microscopic polyangiitis and myeloperoxidase antibody positivity. However, a prospective study of the effect of active sun exposure on the development of AAV has not been performed and the possibilities are hampered by the rarity of the disease.

Our study is the first to investigate the effect of active sun exposure in GCA and PMR. The use of the MISS prospective cohort study to gather information on variation in sun exposure due to differences in behaviour of 14 574 women independent of the assessment of subsequent development of GCA and PMR and the availability of data on potential confounders are strengths of this study. By focusing on a cohort in southern Sweden (≈56° N latitude), we investigate the effect of sun exposure while bypassing the issue of confounding factors with latitudinal gradients. This study design is scalable to other inflammatory rheumatic diseases.

Limitations of this study include the lack of validation of PMR/GCA diagnoses, which were based on registered ICD codes only. A previous survey for this area indicated that only 60% of PMR diagnoses could be verified on structured review of case records by an experienced rheumatologist [50]. Misclassification may thus affect our results. As we did not have data on ethnicity or genetic markers, we cannot assess potential interactions between such factors and sun exposure. Furthermore, the impact of active sun exposure and other factors may vary for PMR and GCA and power was limited for separate analyses of these conditions. Finally, for time-dependent comorbidities, we cannot exclude that early effects of inflammation or healthcare contacts related to early symptoms may influence the registration of such diagnoses.

In conclusion, active sun exposure did not affect the risk of developing GCA or PMR in a large cohort of women in southern Sweden (≈56° N latitude). The aetiology of GCA and PMR likely lies in a complex interplay of aging, environmental triggers and genetic susceptibility, with no evidence supporting a direct effect of active sun exposure.

## Supplementary Material

rkad071_Supplementary_Data

## Data Availability

Raw data are protected by confidentiality laws in Sweden and cannot be shared. All data relevant to the study are included in the article. Please contact the corresponding author.
