# Supplementary material for: Can active sun exposure decrease the risk of giant cell arteritis and polymyalgia rheumatica in women?
Source: Rheumatol Adv Pract. 2023 Aug 18;7(3):rkad071. doi: 10.1093/rap/rkad071 (PMC10477307; doi:10.1093/rap/rkad071)
Supplement: rkad071_Supplementary_Data [file rkad071_supplementary_data.docx]

**Supplementary Table S1:** The ICD10 codes used to identify women with giant cell arteritis, polymyalgia rheumatica, hypertension, hyperlipidaemia, and diabetes.

| Disease | Subitem | ICD10 code |
| --- | --- | --- |
| Outcome |  |  |
|  | Giant cell arteritis with polymyalgia rheumatica | M315 |
|  | Other giant cell arteritis | M316 |
|  | Polymyalgia rheumatica | M353 |
| Hypertension |  | I109 |
| Hyperlipidaemia |  | E78 |
| Diabetes |  | E10, E11 |

**Supplementary Table S2:** Descriptive table of outcome and follow up time stratified by sun exposure level (Sensitivity analysis: ICD-10-code required at two visits)

|  |  | Sun exposure |  |
| --- | --- | --- | --- |
|  | Low | Moderate | High |
| Cohort |  |  |  |
| Total, n | 995 | 8851 | 4728 |
| Outcome |  |  |  |
| PMR, n (%) | 27 (3) | 263 (3) | 109 (2) |
| GCA, n (%) | 8 (1) | 65 (1) | 20 (1) |
| Follow up time in years, mean (SD) | 14.3 (5.5) | 14.2 (5.3) | 13.7 (5.4) |

GCA: giant cell arteritis, PMR: polymyalgia rheumatica, SD: standard deviation.

**Supplementary Table S3:** Univariable hazard ratios and 95% confidence intervals of risk of developing of giant cell arteritis/polymyalgia rheumatica, giant cell arteritis or polymyalgia rheumatica (Sensitivity analysis: ICD-10-code required at two visits)

|  | HR (CI) for GCA/PMR | HR (CI) for GCA | HR (CI) for PMR |
| --- | --- | --- | --- |
| Age |  |  |  |
| 50-54 | REF | REF | REF |
| 55-59 | 2.1 (1.6-2.8) | 2.1 (1.1-3.9) | 2.1 (1.5-2.9) |
| 60-64, | 2.5 (1.9-3.4) | 1.8 (0.9-3.6) | 2.7 (1.9-3.7) |
| 65-69 | 4.2 (3.1-5.6) | 3.0 (1.6-5.6) | 4.5 (3.3-6.1) |
| 70-75 | 4.8 (3.5-6.4) | 4.3 (2.3-8.0) | 4.9 (3.5-6.8) |
| Smoking |  |  |  |
| Never | REF | REF | REF |
| Ever | 0.9 (0.7-1-1) | 0.5 (0.3-0.7) | 1.0 (0.8-1.2) |
| Sun exposure |  |  |  |
| Low | REF | REF | REF |
| Moderate | 1.0 (0.7-1.4) | 0.9 (0.5-1.9) | 1.0 (0.8-1.7) |
| High | 0.8 (0.6-1-2) | 0.6 (0.3-1.3) | 0.9 (0.6-1.4) |
| Obesity |  |  |  |
| BMI ≤30 kg/m^2^ | REF | REF | REF |
| BMI, >30 kg/m^2^ | 1.3 (1.0-1-7) | 0.9 (0.5-1.8) | 1.4 (1.1-1.8) |
| Time dependents |  |  |  |
| Hypertension | 1.9 (1.6-2.4) | 1.2 (0.8-1.9) | 1.9 (1.5-2.3) |
| Hyperlipidaemia | 1.6 (1.2-2.0) | 1.2 (0.7-2.1) | 1.5 (1.2-1.9) |
| Diabetes | 1.2 (0.8-1.6) | 0.9 (0.4-2.0) | 1.3 (0.9-1.8) |

HR: hazard ratio, CI: confidence interval, GCA: giant cell arteritis, PMR: polymyalgia rheumatica, BMI: body mass index, REF: reference level

**Supplementary Table S4:** Multivariable hazard ratios and 95% confidence intervals of risk of developing giant cell arteritis/polymyalgia rheumatica, giant cell arteritis or polymyalgia rheumatica, adjusted for diabetes, hyperlipidaemia, hypertension, smoking, obesity and stratified by age (Sensitivity analysis: ICD-10-code required at two visits)

|  | HR (CI) for GCA/PMR | HR (CI) for GCA | HR (CI) for PMR |
| --- | --- | --- | --- |
| Sun exposure |  |  |  |
| Low | REF | REF | REF |
| Moderate | 1.3 (0.9-1.8) | 1.2 (0.6-2.4) | 1.4 (1.0-2.2) |
| High | 1.4 (0.9-2.1) | 0.9 (0.4-2.0) | 1.6 (1.0-2.4) |
| Obesity |  |  |  |
| BMI ≤30 kg/m^2^ | REF | REF | REF |
| BMI, >30 kg/m^2^ | 1.2 (0.9-1.5) | 0.9 (0.4-1.7) | 1.2 (0.9-1.6) |
| Smoking |  |  |  |
| Never | REF | REF | REF |
| Ever | 1.1 (0.9-1.3) | 0.6 (0.4-0.8) | 1.2 (1.0-1.5) |
| Time dependents |  |  |  |
| Hypertension | 1.5 (1.2-1.6) | 1.1 (0.7-1.7) | 1.5 (1.2-1.9) |
| Hyperlipidaemia | 1.2 (0.9-1.6) | 1.1 (0.6-2.1) | 1.2 (0.9-1.5) |
| Diabetes | 0.8 (0.6-1.2) | 0.8 (0.3-1.8) | 0.9 (0.6-1.3) |

HR: hazard ratio, CI: confidence interval, GCA: giant cell arteritis, PMR: polymyalgia rheumatica, BMI: body mass index, REF: reference level. Age included as a stratified variable and not shown.
